# Polycaprolactone Scaffolds Fabricated via Bioextrusion for Tissue Engineering Applications

**DOI:** 10.1155/2009/239643

**Published:** 2009-09-08

**Authors:** Marco Domingos, Dinuccio Dinucci, Stefania Cometa, Michele Alderighi, Paulo Jorge Bártolo, Federica Chiellini

**Affiliations:** ^1^Department of Chemistry & Industrial Chemistry, University of Pisa, 56126 Pisa, Italy; ^2^Center for Rapid and Sustainable Product Development, Polytechnic Institute of Leiria (IPL), 2414-016 Leiria, Portugal

## Abstract

The most promising approach in Tissue Engineering involves the seeding of porous, biocompatible/biodegradable scaffolds, with donor cells to promote tissue regeneration. Additive biomanufacturing processes are increasingly recognized as ideal techniques to produce 3D structures with optimal pore size and spatial distribution, providing an adequate mechanical support for tissue regeneration while shaping in-growing tissues. This paper presents a novel extrusion-based system to produce 3D scaffolds with controlled internal/external geometry for TE applications.The BioExtruder is a low-cost system that uses a proper fabrication code based on the ISO programming language enabling the fabrication of multimaterial scaffolds. Poly(*ε*-caprolactone) was the material chosen to produce porous scaffolds, made by layers of directionally aligned microfilaments. Chemical, morphological, and in vitro biological evaluation performed on the polymeric constructs revealed a high potential of the BioExtruder to produce 3D scaffolds with regular and reproducible macropore architecture, without inducing relevant chemical and biocompatibility alterations of the material.

## 1. Introduction

Scaffolds are support structures used in tissue engineering to provide the three-dimensional growth of cells in an organized way. They are often critical, both in vitro and in vivo, as they serve some of the following purposes [1–4]: allow cell attachment, proliferation, and differentiation, deliver and retain cells and growth factors, enable diffusion of cell nutrients and oxygen, and enable an appropriate mechanical and biological environment for organized tissue regeneration. 

The ideal scaffold should be biocompatible (not eliciting adverse host tissue response), biodegradable into nontoxic products with a controlled degradation rate, and possess an optimized micro/macrostructural feature capable of guarantying simultaneously both adequate vascularisation and sufficient mechanical strength to withstand stresses in the host tissue environment. An adequate surface topology must also be achieved to promote cell-cell and cell-matrix interactions.

The design, production, and characterization of tissue engineering constructs are therefore demanding tasks in regenerative medicine, namely, for load bearing tissues such as bone and cartilage. Large pores and highly porous structures are required to promote in vivo direct osteogenesis (allowing vascularisation and proper oxygen supply to the cells), while smaller pores result in osteochondral ossification. The interconnectivity of such pores is also significant because of cell migration, and therefore vascularisation will be inhibited if pores are not interconnected, even if the structures present an elevated porosity [5].

Although highly porous structures enhance bone tissue ingrowth, it also leads to a reduction in terms of mechanical properties that may compromise the structural stability of the scaffold. An upper limit for pore size and porosity may than be established, based both on mechanical constraints and host tissue pore dimensions [6]. The strength of a scaffold is primarily dependent of the material bulk properties, but the fabrication method also plays an important role conferring the opportunity to manipulate overall porosity. 

The importance of pore size and spatial distribution for effective cell growth and tissue regeneration was underlined by Oh et al. [7]. He reported an ideal pore size range of 380–405 *μ*m for chondrocytes and osteoblasts, 186–200 *μ*m for fibroblasts, and 290–310 *μ*m for bone formation. 

Several techniques were developed to produce 3D matrices suitable for tissue engineering, including nonconventional techniques. These methods include Solvent casting/salt leaching, Phase separation, Gas Foaming freeze drying, among others. Despite being possible to control the pore size and shape by changing the process parameters, the interconnectivity and spatial distribution of the pores are still very poor. These limitations result in partially interconnected and randomly dispersed pores leading to an insufficient vascularisation and tissue ingrowth. 

Additive fabrication processes represent a new group of nonconventional techniques recently introduced in the medical field [8]. The main advantages of these techniques are the capacity to rapidly produce very complex 3D models, the ability to use various raw materials, and high reproducibility. In the tissue engineering field, additive technologies have been used to produce scaffolds with customized external shape and predefined internal morphology, allowing a good control of pore size and pore distribution [1]. 

Extrusion-based strategies to produce complex and highly interconnected pore structures and the viability of such structures to accommodate cells enabling its proliferation have been explored by several researchers [9–13]. However, during the fabrication process, materials undergo phase change phenomena (solid-liquid-solid) under relatively high temperature and pressure, on top of being subject to relatively high shear rates during the extrusion process. These phase change phenomena and the processing conditions considered for each application may induce chemical and physical transformations in a material. As a consequence, the biocompatibility characteristics of the initial material can be altered during its fabrication process. 

This paper investigates the use of a novel BioExtruder system to produce polymeric scaffolds for tissue engineering applications, by examining the effect of the processing conditions in terms of the chemical-physical properties and biocompatibility of the processed polymeric material.

**Figure 1:**
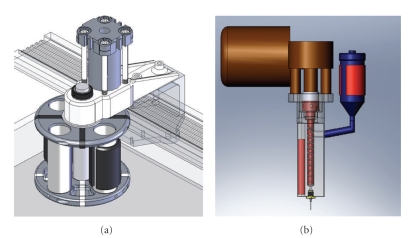
(a) Multimaterial extrusion system. (b) Single-material extrusion system.

The BioExtruder is an additive biomanufacturing system under development for tissue engineering applications [14, 15]. It is a highly reproducible and low-cost system enabling the controlled definition of pores into the scaffold to modulate mechanical strength and molecular diffusion, as well the fabrication of multimaterial scaffolds. It comprises two different deposition systems: one rotational system for multimaterial deposition acted by a pneumatic mechanism and another one for a single material deposition that uses a screw to assist the deposition process (Figure 1). 

The rotational system has four reservoirs, two with temperature control and two without. A large number of nozzle diameters ranging from 0.1 to 1 mm can be used. The information flowchart to produce scaffolds for tissue engineering through this BioExtruder system comprises three main steps. The first one is the generation of the corresponding computer solid model either directly by Computer Aided Design (CAD) software or by using the data derived from the currently available imaging techniques (magnetic resonance or computer tomography). The computer solid model is then tessellated as a stereolithography (STL) file, which is currently the standard file for faceted models. For multimaterial scaffolds, the BioExtruder system uses the PLY (Polygon File Format) file format describing the model through a list of flat polygons. In this format, it is possible to attribute different properties and characteristics to each polygon. Finally, the STL or PLY model is mathematically sliced into thin layers (sliced model). Each layer is then physically reproduced by the BioExtruder system.

**Figure 2:**
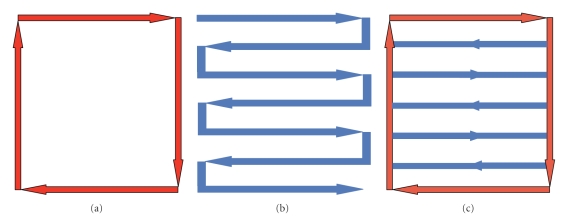
Deposition strategies: (a) contour, (b) raster, (c) raster and contour.

Three scanning strategies were implemented for the deposition process (Figure 2): contour, raster, and contour and raster.

The deposition code, developed in Matlab (The MathWorks, Inc.), is based on the ISO programming language, commonly used to control Computer Numerical Control (CNC) machines.

In addition, it should be stressed the fact that tissues are organized in different layers in the human body, with different physical and biological functions, and hence it becomes important to produce scaffolds that can mimic this functional hierarchy. The Bioextruder opens new perspectives regarding the fabrication of these so called functionally graded scaffolds by enabling the deposition of different materials at specific layers or by manipulating porosity and pore size/shape through a set of process parameters.

## 2. Materials and Methods

### 2.1. Materials

Poly (*ε*-caprolactone) with Mw 50.000 (CAPA 6500) in the form of 3 mm pellets was obtained from Perstorp Caprolactones (Cheshire, United Kingdom). Chloroform used for GPC analysis was purchased from Sigma Aldrich. 

### 2.2. Scaffolds Design and Fabrication

A BioExtruder device, developed at the Polytechnic Institute of Leiria (I.P.L., Pt), was used to fabricate the 3D porous scaffolds. Briefly, rectangular prisms, measuring 40 (length) × 40 (width) × 8 mm (height) with an average porosity of ~76%, were initially designed in a CAD software (SolidWorks, Dassault Systèmes S.A.). The STL file format was then transferred to the BioExtruder slice generator software where it was automatically sliced. This routine consists of a slicing algorithm that slices the STL model into a number of 2D layers of predefined thickness to generate the contours of the model (SLI file). The deposition strategy (raster deposition strategy), scanning velocity, and filament distance for each layer were directly programmed through the BioExtruder scanning deposition generator routine, which was developed based on the ISO programming language for CNC machines. A 0/90 lay-down pattern was implemented in order to produce a honeycomb-like pattern of fully interconnected square pores. The information was then sent to the extrusion equipment where the 3D structures were plotted in a layer-by-layer fashion by applying an extrusion pressure and screw rotation velocity of 6 bar and 30 rpm, respectively, enabling the extrusion of the material through a 300 *μ*m nozzle. The liquefier and extrusion chamber temperature were set at 70°C, and ambient temperature remained at 25 ± 2°C. The 3D structures were then removed from the building platform and used for chemical and biological characterization as described in the following sections.

### 2.3. Chemical Analysis

#### 2.3.1. Gel Permeation Chromatography

The molecular weight and the polydispersity of PCL samples were obtained by gel permeation chromatography (GPC) using chloroform as the eluent at a flow rate of 1.0 mL/min. The injection volume was usually 50 *μ*L of stock solutions (0.5% w/v). A Jasco PU-1580 HPLC liquid chromatograph connected to Jasco 830-RI and Perkin-Elmer LC-75 spectrophotometric (*λ* = 260 nm) detector, equipped with two PLgel 5 *μ* mixed-D columns, was used. The calibration curve was established by using nine monodispersed polystyrene standards (Perkin-Elmer) with molecular weight of 300, 233, 83, 50, 19, 4, 2.1, 0.8, and 0.5 KDa, respectively.

#### 2.3.2. Differential Scanning Calorimetry

The thermal properties were determined by differential scanning calorimetry (DSC) using a Mettler TA 400 system instrument consisting of DSC-30 differential scanning calorimeter. DSC samples of 6 mg were weighed in 40 *μ*L aluminium pans; an empty pan was used as reference. Measurements were carried out under nitrogen atmosphere (nitrogen flow rate of 80 mL/min). The sample was submitted to a 1st heating from 30 to 90°C, at a heating rate of 10°C/min, and then cooled from 90 to −100°C at a cooling rate of 10°C/min, followed by 4 minutes of isotherm. The 2nd heating scan was run from −100 to 90°C at a heating rate of 10°C/min. The melting temperatures (*T*_*m*_) were obtained at the peak of the melting endotherms, while the glass transition temperatures (*T*_*g*_) were taken at the inflection point of the specific heat capacity. The enthalpies of fusion (∆*H*_*f*_) were directly obtained from the areas under the peaks. All values were taken from the thermograms relevant to the second heating cycle. Indium and gallium samples were used as calibration standards.

### 2.4. Scaffold Morphology

#### 2.4.1. Scanning Electron Microscopy

Morphological analysis of the 3D structures was carried out using a scanning electron microscope (SEM, Jeol LSM 5600LV, Tokyo, Japan) to visualize and evaluate the physical integrity of the material filaments and layers, as well to understand if the previously defined pore geometry and size were maintained constant during production. 

#### 2.4.2. Atomic Force Microscopy

A commercial AFM instrument (Multimode microscope working with a Nanoscope IV controller, Veeco Instr. Santa Barbara Ca. USA) was used to evaluate the surface topography of the extruded filaments. The AFM is equipped with a PicoForce stage allowing for closed-loop scans in the *Z* direction (J-type scanner) and thus endowing a precise and reliable movement along the *z* axis, useful during elasticity measurements. The cantilever used for imaging, an RTESP probe made of silicon, is 125 *μ*m long and presents a typical oscillation frequency of 32 KHz.

### 2.5. Biological Tests

#### 2.5.1. Materials

Cell line BALB/3T3 Clone A31 mouse embryo fibroblasts (CCL163) was obtained from American Type Culture Collection (ATCC) and propagated as indicated by the supplier. Dulbecco's Modified Eagles Medium (DMEM), 0.01 M pH 7.4 phosphate buffer saline without Ca^2+^ and Mg^2+^ (PBS_1_), Calf Serum (CS), trypsine/EDTA, glutamine, and antibiotics (penicillin/streptomycin) were purchased from GIBCO Brl. Cell proliferation reagent WST-1 was purchased from Roche Diagnostic. PhalloidinAlexa488 and 4′,6-diamidino-2-phenylindole (DAPI) were purchased from Invitrogen (NewYork, NY, USA). Tissue culture grade disposable plastics were obtained from Corning Costar.

#### 2.5.2. Cytotoxicity Tests on Extracts

To assess the cytotoxicity of possible substances that could leach from both raw and processed PCL, 0.2 g of each material was placed in 1 mL of DMEM supplemented with 10% Calf Serum, 4 mm L-Glutammine, and 100 U/mL : 100 *μ*g/mL penicillin : streptomycin for 48 hours at 37°C in an enriched 5% CO_2_ atmosphere. The medium containing the extracts was tested undiluteed and diluted at a volume ratio of 1 : 1 and 1 : 4 using the complete culture medium. Balb/c 3T3 Clone A31 cells were seeded at a density of 1 × 10^3^/well in a 96-well plate and allowed to proliferate for 24 hours. Then the culture media was changed with the DMEM containing the extracts, and cells were allowed to proliferate for further 24 hours at 37°C in a 5% CO_2_ enriched atmosphere. Cells incubated with complete DMEM and wells containing only complete DMEM were used as controls. At the end of the exposure time, cell viability was measured using WST-1 tetrazolium salt. Absorbance was measured at 450 nm, and values relative to control were reported. Experiments were performed in triplicates. 

#### 2.5.3. Cell Adhesion and Proliferation onto PCL Scaffolds

To investigate the ability of the prepared PCL scaffolds to support cell adhesion and proliferation, samples were sterilized with 70% ethanol/water solution for 24 hours, washed extensively with PBS 0.01 M pH 7.4 and exposed to U.V light for 40 minutes. 

Then cells were seeded at an appropriate density (3 × 10^4^cells/mL) directly onto the PCL scaffolds and allowed to proliferate for 6 days. At the end of the incubation time samples were analyzed for cell proliferation by means of WST-1 tetrazolium salts as previously described. 

For cell morphology investigations after six days of culture samples were fixed with 3.8% paraformaldehyde solution in PBS 0.01 M pH 7.4 for 1 hour and permeabilized with Tritox X-100 for 15 minutes to enhance dyes binding to cellular structure. A number of scaffolds were incubated with toluidine blue for 1 hour under gently stirring and extensively washed with PBS in order to stain cells for bright field observation. 

Cell morphology was investigated also by confocal laser scanning microscopy (CLSM) and scanning electron microscopy (SEM). For CLSM after the permeabilization step with Triton X-100, samples were incubated with a PBS 0.01 M solution of DAPI and phalloidin-Alexa633 for 45 minutes at room temperature. After dyeing incubation, scaffolds were washed with PBS and cut into slices before mounting on a glass slide and sealing with resin for microscopic observation.

A Nikon Eclipse TE2000 inverted microscope equipped with an EZ-C1 confocal laser (Nikon, Japan) and Differential Interference Contrast (DIC) apparatus and a 60X oil-immersion objective were used to analyze the samples. A 405 nm laser diode (405 nm emission) and Argon Ion Laser (488 nm emission) were used to excite, respectively, DAPI and FITC fluorophores. Images were captured with Nikon EZ-C1 software with identical settings for each sample. Images were further processed with GIMP (GNU Free Software Foundation) Image Manipulation Software and merged with Nikon ACT-2U Software. For SEM analysis samples were fixed with solution of 2.5% glutaraldehyde in PBS 0.01 M for 1 hour at room temperature and dehydrated by the use of a series of ethanol solutions (25%, 50%, 70% and 100% v/v). Afterward constructs were air dried overnight at room temperature and sputter coated with gold and analyzed by a JEOL LSM5600LV scanning electron microscope. Statistical analysis is the following for every test, the data are expressed as means plus or minus the standard deviation (*n* = 3). The statistical analysis was performed with Students' *t*-test at a 0.05 level.

## 3. Results and Discussion

### 3.1. Scaffold Design and Fabrication

**Figure 3:**
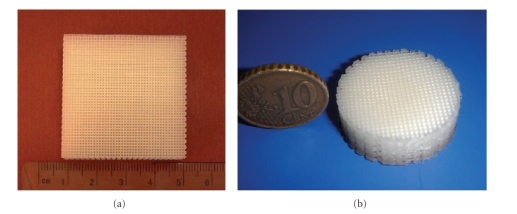
Square and disc shape scaffolds produced by the BioExtruder.

PCL scaffolds were designed and produced with different external geometries (squared and circular shapes) and fully interconnected square pores (Figure 3). One single lay-down pattern 0/90° deposition strategy was adopted to generate the 3D structures. Process parameters, as shown in Table 1, were set according to the material properties and scaffold geometry.

**Figure 4:**
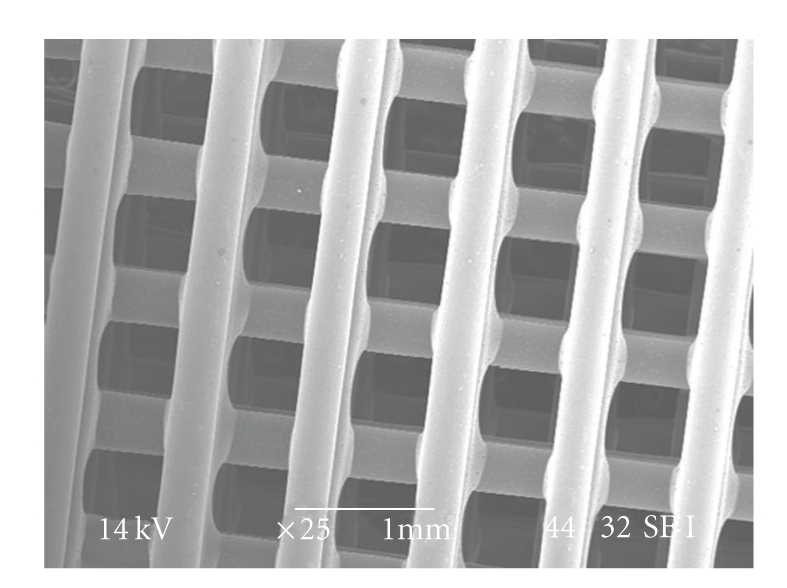
Scanning Electron Microscopy (SEM) micrograph of a scaffold (top view).

A morphological evaluation of the structures was carried out under Scanning Electron Microscopy (SEM). The scaffolds produced with the BioExtruder present a well-defined internal geometry with square interconnected pores of regular dimensions (~600 × 600 *μ*m) and uniform distribution, when viewed from the *Z* direction of the fabrication process (Figure 4). The extruded filaments show a regular circular geometry with ~350 *μ*m diameter, according to the nozzle tip used (300 *μ*m).

**Figure 5:**
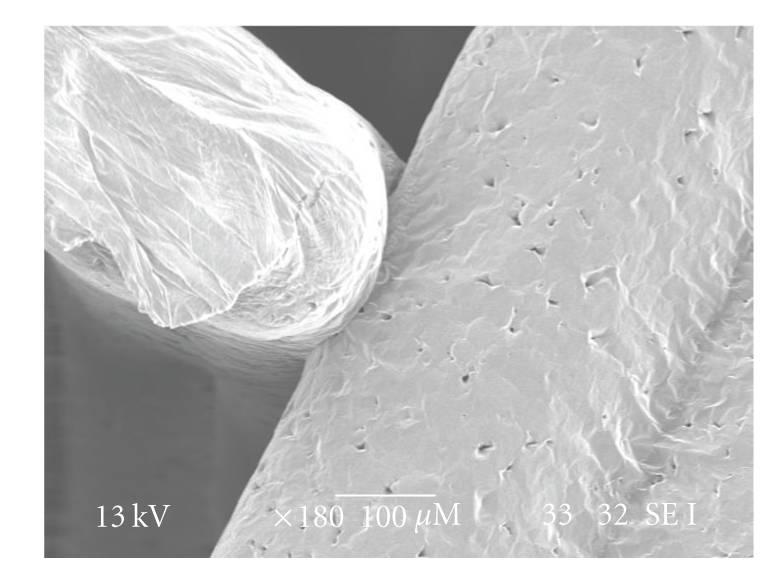
SEM micrograph of filament adhesion detail.

Adhesion between adjacent layers appeared to be good, though mechanical tests need to be performed in the future to quantitatively validate these results (Figure 5). The microscopic analysis also confirmed the acceptable level of material homogeneity.

Scaffold porosity was determined according to the following equation:

(1) $$\text{Porosity}=1-\frac{\rho^{*}}{\rho_{\text{sub}}},$$

where ρ* is the density of the cellular structure, and *ρ*_sub_ is the density of the original substance. The produced scaffolds porosity was found to be ~76%.

**Figure 6:**
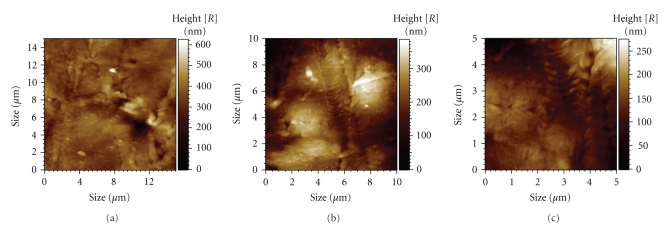
3D images of the scaffolds surface topography obtained with AFM. (a) area 1; (b) area 2; (c) area 3.

The microporosity of a scaffold is an important parameter to determine both cell-scaffold and cell-cell interactions during the adhesion and proliferation process on the surface of the scaffolds. It is also important to produce filaments neither too flat nor too rough. If the surface is too rough, cells adhering to the filaments might not be able either to develop distinct focal adhesion plaques or bridge the irregularities and establish cell-cell interactions. Moreover, the sharpness of the surface can physically damage the cell. Results of AFM analysis performed on three different areas of the scaffold filaments are illustrated in Table 2 and Figure 6. 

As previously mentioned, porosity and pore size/shape plays an important role both on the mechanical and biological performance of tissue engineering scaffolds. The structures produced with the BioExtruder present an elevated porosity and large interconnected pores that are capable to fulfil the requirements for appropriate vascularisation and tissue growth. Depending upon the tissue to be regenerated, the BioExtruder offers the possibility to modulate the biomechanical properties of the scaffolds, applying different process and design parameters.

### 3.2. Process Viability

Chemical and biological analyses were performed to validate the bioextrusion process to produce PCL scaffolds for tissue engineering applications.

Potential degradation or chemical/physical modifications of the polymeric material, caused by the extrusion process, was investigated by Gel Permeation Chromatography (GPC) and Differential Scanning Calorimetry (DSC). The GPC results, presented in Table 3, showed no particular modifications in the molecular weight (*M*_*n*_) and polydispersity index (*M*_*w*_/*M*_*n*_), indicating a great stability of the polymer under the selected fabrication conditions.

Through DSC analysis, it was possible to confirm that the crystalline fraction of PCL did not rise significantly when the polymer was processed via bioextrusion. The values for nonprocessed and processed PCL ranged between 50% and 52%, respectively. The thermodynamics characteristics of both PCL samples are indicated in Table 4. 

### 3.3. Biological Evaluation

#### 3.3.1. Cytotoxicity

**Figure 7:**
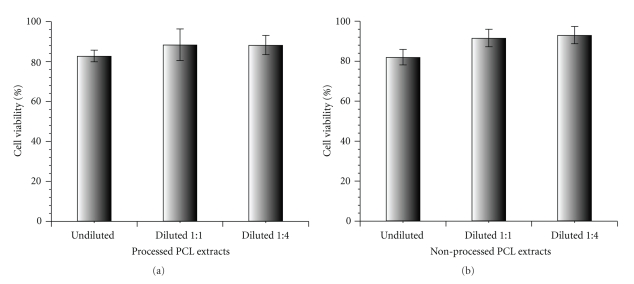
WST-1 cell proliferation assay performed on PCL extracts in solution with 3T3/A31 fibroblasts: (a) processed PCL extracts; (b) nonprocessed PCL extracts.

Materials intended to be used for tissue engineering applications should not release any agent that may be cytotoxic. To know whether the prepared PCL scaffolds extracts might be harmful to cells, the fibroblast cell line balb/c 3T3 Clone A31 was cultured in the presence of the extractables of the unprocessed and bioextruded PCL over 24 hours at 37°C, and WST-1 assay was carried out to evaluate their potential cytotoxicity. Aqueous extracts of the investigated samples, undiluted, diluted 1 : 1 and 1 : 4, were used. The result of this study, shown in Figure 7, revealed that no significant release of cytotoxic compound occurs from the PCL material either before and after the bioextrusion process and that cells not only remained viable but also proliferated similar to the control also in the case of the undiluted extract.

#### 3.3.2. Cell Adhesion and Proliferation onto PCL Scaffolds

**Figure 8:**
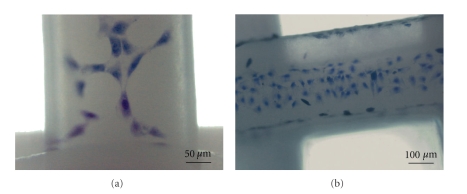
Bright field micrographs of mouse embryo fibroblasts stained with toluidine blue after six days of culture onto PCL scaffolds.

A preliminary biological evaluation of the prepared PCL scaffolds to sustain cell adhesion and proliferation was carried out by using the fibroblast cell line balb/c 3T3 Clone A31. Quantitative evaluation of cell proliferation onto the bioextruded PCL scaffold was performed after 6 days of static culture by means of WST-1 tetrazolium salt. Results highlighted a cell proliferation on the investigated sample in the range of 15% in respect to the cells grown on tissue culture polystyrene (TCPS), used as control. This fairly low proliferation is probably due to the large pore size of the prepared scaffolds that enables a large number of cells not to settle on the filaments. Nevertheless, the parallel investigations of cell morphology carried out by optical microscopy revealed a significant proliferation of the cells on the surface of the filaments with a clear spreading along them and good morphology (Figure 8). 

**Figure 9:**
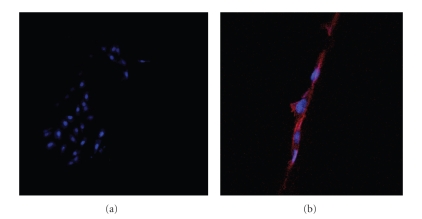
Confocal micrographs of mouse embryo fibroblasts stained with DAPI (nuclei) and Phalloidin-Alexa633 (cytoskeleton) after 6 days of culture.

CLSM investigations confirmed this observation, highlighting a general alignment of the actin fibers of cells cytoskeleton along the scaffold filaments and a nucleus with normal morphology (Figure 9).

**Figure 10:**
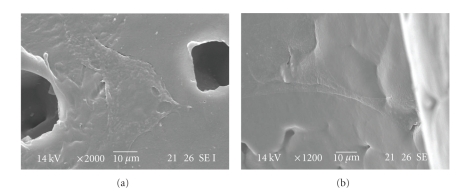
SEM micrographs of cells adhesion and spreading on the external and internal surface of the PCL scaffolds.

In addition, SEM analysis confirmed the adhesion of cells both on the external and internal surface of the scaffolds (Figure 10). These preliminary results appear very promising, and further experiments will be aimed at determining the cell proliferation and morphology onto the bioextruded scaffolds after longer period of culture. 

## 4. Conclusions

The BioExtruder system was investigated and successfully used to produce 3D porous scaffolds with fully interconnected pores of regular geometry and dimension.

Results from chemical analysis demonstrated that no significant alterations were introduced in the material neither by the high temperatures or shear forces involved in the melt/extrusion process. Cytotoxicity screening tests show a high percentage of cell proliferation (>80%) whenever 3T3/A31 fibroblasts were placed in contact with processed material extracts, which seem to indicate that no substantial quantities of toxic compounds were released from the bioextruded PCL scaffold. 

After 6 days of static cell culture, cells were able to adhere and proliferate on the prepared scaffolds, showing an alignment along the filaments. A qualitative cell adhesion along with AFM evaluation allowed us to validate the surface topography of the constructs, demonstrating that it is possible to obtain structures with adequate topography capable of promoting cell-surface and cell-cell interactions through bioextrusion. 

New deposition strategies are currently being explored in order to obtain new geometries and consequently evaluate their influence on both the mechanical and biological properties of the structures. 

In conclusion, these preliminary results not only confirm the high potential of the BioExtruder system to produce PCL scaffolds for bone tissue engineering but also open the possibility to explore the design and fabrication of multimaterial scaffolds that can meet the functional and biological requirements of the living tissues, through the use of the rotational deposition system. This system, as mentioned before, thanks to the four material chambers, two with temperature control and two without activated by a pneumatic mechanism, will enable further experiments for the extrusion of 3D bioactive scaffolds that can incorporate growth factors or living cells. 

## Figures and Tables

**Table 1:** Process conditions.

Process conditions	
Reservoir temperature	70°C
Reservoir pressure	6 bar
Screw velocity	30 rpm
Extrusion chamber temperature	70°C
Extrusion nozzle diameter	0.30 mm
Scanning velocity	8 mm/s
Nozzle/platform distance	0.2 mm

**Table 2:** AFM statistic results.

Statistics	Scan area 1	Scan area 2	Scan area 3
Scan area (*μ*m × * μ*m)	100	25	225
Root mean squared of	213.5	140.2	360.1
pixel values (nm)			
Standard deviation	65.8	43.0	48.8

**Table 3:** Molecular weight distribution of processed and nonprocessed PCL.

PCL Sample	Sample Code	*M*_*n*_	*M*_*w*_	*M*_*w*_/*M*_*n*_
Processed	MD1	89900	123000	1.37
Nonprocessed	MD	89500	127500	1.42

**Table 4:** Thermodynamics characteristics of PCL during second heating scan.

PCL Sample	Sample Code	*T*_*g*_ (°C)	*T*_*m*_ (°C)	Δ*H*_*m*_(J/g)
Processed	MD1	−60. 8	56.1	69.7
Nonprocessed	MD	−63. 5	57.0	72.0
